# Effect of Morphology Modification of BiFeO_3_ on Photocatalytic Efficacy of P-g-C_3_N_4_/BiFeO_3_ Composites

**DOI:** 10.3390/ijms25094948

**Published:** 2024-05-01

**Authors:** Abubakar Usman Katsina, Diana-Luciana Cursaru, Dănuţa Matei, Sonia Mihai

**Affiliations:** 1 Faculty of Petroleum Technology and Petrochemistry, Petroleum—Gas University of Ploiesti, 100680 Ploiesti, Romania; aukatsina.chm@buk.edu.ng (A.U.K.); dianapetre@upg-ploiesti.ro (D.-L.C.); danuta.matei@upg-ploiesti.ro (D.M.); 2 Department of Pure and Industrial Chemistry, Bayero University, Kano PMB 3011, Nigeria

**Keywords:** wastewater treatment, perovskite bismuth ferrite (BiFeO_3_), graphitic carbon nitride (g-C_3_N_4_), morphology-controlled synthesis, heterojunction photocatalyst, photocatalytic degradation, non-metallic doping, urea-assisted hydrothermal synthesis, rhodamine B (RhB)

## Abstract

This current study assessed the impacts of morphology adjustment of perovskite BiFeO_3_ (BFO) on the construction and photocatalytic activity of P-infused g-C_3_N_4_/U-BiFeO_3_ (U-BFO/PCN) heterostructured composite photocatalysts. Favorable formation of U-BFO/PCN composites was attained via urea-aided morphology-controlled hydrothermal synthesis of BFO followed by solvosonication-mediated fusion with already synthesized P-g-C_3_N_4_ to form U-BFO/PCN composites. The prepared bare and composite photocatalysts’ morphological, textural, structural, optical, and photocatalytic performance were meticulously examined through various analytical characterization techniques and photodegradation of aqueous rhodamine B (RhB). Ellipsoids and flakes morphological structures were obtained for U-BFO and BFO, and their effects on the successful fabrication of the heterojunctions were also established. The U-BFO/PCN composite exhibits 99.2% efficiency within 20 min of visible-light irradiation, surpassing BFO/PCN (88.5%), PCN (66.8%), and U-BFO (26.1%). The pseudo-first-order kinetics of U-BFO/PCN composites is 2.41 × 10^−1^ min^−1^, equivalent to 2.2 times, 57 times, and 4.3 times of BFO/PCN (1.08 × 10^−1^ min^−1^), U-BFO, (4.20 × 10^−3^ min^−1^), and PCN, (5.60 × 10^−2^ min^−1^), respectively. The recyclability test demonstrates an outstanding photostability for U-BFO/PCN after four cyclic runs. This improved photocatalytic activity exhibited by the composites can be attributed to enhanced visible-light utilization and additional accessible active sites due to surface and electronic band modification of CN via P-doping and effective charge separation achieved via successful composites formation.

## 1. Introduction

The prevalent pollution of the environment stems from an increased population, rapid industrial expansion, and energy scarcity [1]. It poses colossal threats to human health and susceptible ecosystems, leading to an intensifying looming global warming concern [2]. Numerous measures have been employed to tackle these environmental-related challenges using eco-friendly techniques, including photodegradation of aqueous contaminants in industrial wastewater treatment [3,4,5,6,7]. Scholars in environmental remediation and sustainable energy generation appreciate the profound practicality of semiconductor photocatalysis technology, owing to its light absorption ability to generate electron–hole pairs for surface chemical reactions [8,9]. Diverse classes of recalcitrant contaminants, including dye effluents [10], phenols [11], pharmaceuticals [12], and personal hygiene products [13], have been reported to be completely decomposed by visible-light active semiconductor photocatalysts. However, the applicability of the commonly reported TiO_2_ and other wide bandgap semiconductors is confined to only the ultraviolet shortwave spectral region [14], lacking efficient solar energy exploitation that fundamentally relies on light absorption in longer wavelength regions, including visible light and infrared. Thus, exploring other narrow-band optical gap semiconductors with superior performance for photocatalytic applications utilizing the visible spectrum is essential.

Bismuth-containing compounds, including BiFeO_3_ (BFO), BiMnO_3_, Bi_2_WO_6_, BiOBr, and BiVo_4,_ are known to be semiconductor photocatalysts that exhibit favorable photoresponsive traits under visible light irradiation due to their narrow band gap and high chemical stability [15,16]. Despite the extensive reports on BiOBr and BiVO_4_ photocatalysts, BFO garnered widespread attention owing to its unique room-temperature multiferroic and piezoelectric properties in a single-phase rhombohedral structure [17,18]. However, the photocatalytic prowess of pristine BFO semiconductors is noticeably low in the reported literature. Several studies established that rapid electron–hole recombination, small accessible active sites, and poor stability are the principal factors leading to its inefficient photocatalytic performance [19]. Numerous strategies, including heterojunction formation [20,21], doping [22,23], morphology engineering [24,25], etc., have been utilized to enhance the efficiency of BFO catalysts by providing better charge carrier separation and more accessible active sites.

In the course of BFO-based heterojunction development, the BFO is integrated with other semiconductor catalysts to form a heterostructured composite, aiming to improve the efficiency of charge carrier separation and optical activity in the visible spectrum [26]. Several semiconductors, including polymeric graphitic carbon nitride (g-C_3_N_4_), have been identified as attractive co-catalyst materials with BFO and other oxide semiconductors [27,28]. The g-C_3_N_4_ (CN) possesses captivating characteristics such as exceptional chemical and thermal resilience, optical characteristics, and non-corrosive nature that are suitable for photocatalytic applications [29,30]. Nonetheless, the practical application of both bare and composite structures of BFO is hindered by the fast recombination of charge carriers due to the difficulty of transferring the separated charges [31]. Infusion of non-metals such as oxygen (O), phosphorous (P), sulfur (S), and nitrogen (N) has been acknowledged as one of the suitable approaches to improving the practical usage of CN materials through band structure modification [32,33]. For example, in our recently published work [34], we have reported a superior visible light photocatalytic activity of Pt@BFO/O-CN towards RhB degradation. Similarly, Chowdhury et al. [35] have investigated the interactive effects of P-doped CN-supported BiVO_4_ heterojunction on the photocatalytic oxidation of synthetic colorants under visible-light irradiation by their improved optoelectronic properties. Long et al. [36] reported a design of S-doped CN and BiPO_4_ hybrid composite heterostructures to enhance hydrogen’s visible light photocatalytic evolution.

Moreover, the morphological configuration and particle size of BFO materials have been stipulated to play a substantial role in their photocatalytic performance [37,38]. Again, synthesis routes such as hydrothermal (HT), sol-gel (SG), and co-precipitation (CP) are deemed essential in tailoring the BFO structural morphology, consequently leading to an adjustment in their properties and performance in various photocatalytic applications [39]. For example, using HT, SG, and CP synthesis methods, Chien et al. [40] effectively obtained coralloid, rod-like, and sheet-like BFO morphological structures. The SG-synthesized rod-like BFO sample outperformed both the HT-coral-like and CP-sheet-like BFO samples in the photodegradation of phenol, illustrating the likely influence of the synthesis route on photocatalytic performance. Likewise, Djabotai et al. [41] have rationally engineered the morphology of BFO morphological nanostructures, influencing the surface’s electron–hole separation, photovoltage characteristics, photogenerated current density, and oxygen production activity.

In the scope of this work, we successfully fabricated a phosphorous-doped g-C_3_N_4_ (PCN) supported heterostructured composites incorporating morphology-tailored architectures of bismuth ferrite (BFO) via a facile sequential methanol sonication and calcination. Urea-free and urea-assisted hydrothermal synthesis routes were employed to prepare BFO and U-BFO catalysts, respectively. Meanwhile, P-doping of PCN was achieved via CH_3_OH-assisted sonication of dibasic ammonium phosphate and CN mixtures, followed by thermal polymerization of the homogenized blend. The texture, electronic, and morphological configurations of CN were significantly engineered by both P-doping and BFO (U-BFO) incorporation in the structure of CN, which led to improved photocatalytic activity and stability towards RhB degradation. The combined effect of doping and morphology-tailored heterojunction formation on the activity of the composites was investigated. The improved photodegradation performance can be attributed to the enhanced separation efficiency of photo-excited charge carriers due to improved visible-light harvesting and enriched synergistic active sites. A four-cycle recyclability test confirmed the photostability and economic feasibility of U-BFO/PCN composites.

## 2. Results and Discussion

### 2.1. Materials Characterization

#### 2.1.1. Fourier-Transform Infrared (FT-IR)

**Figure 1:**
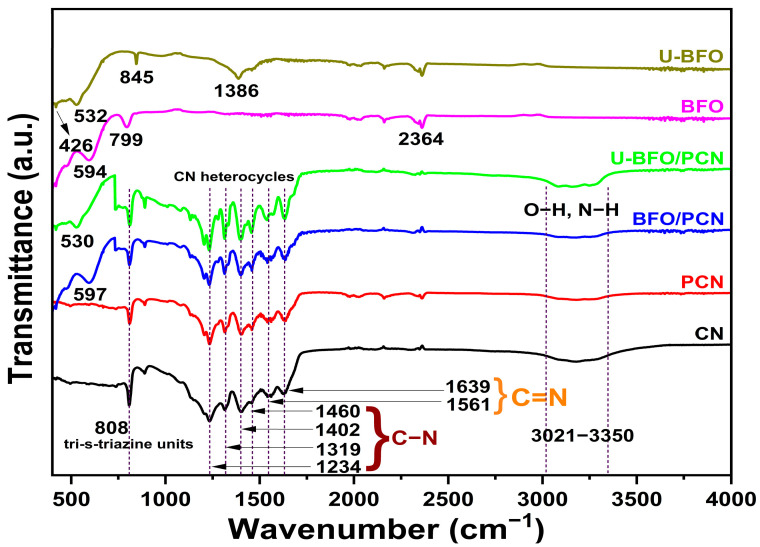
FT-IR spectral patterns of pristine and composite photocatalysts.

Figure 1 shows the FTIR spectra revealing the different functional groups and chemical bonds of all the as-prepared samples. The spectra of CN and PCN reveal a pronounced absorption peak at 808 cm^−1^ attributable to the breathing mode and bending vibration of tri-s-triazine motifs [42]. The robust vibration bands in the range of 1100–1700 cm^−1^ are related to the stretching modes of C–N heterocycles, including the aromatic C–N stretching vibration bands at 1234, 1319, 1402, and 1460 cm^−1^ [43,44], and the C=N stretching vibrational peaks found at 1561 and 1639 cm^−1^ [45]. The broad absorption band observed between 3021 and 3350 cm^−1^ can be indexed to the N–H and O–H stretching vibrations from –NH and adsorbed H_2_O, respectively [46,47]. The FTIR spectral bands in BFO and U-BFO samples both show the presence of Bi–O stretching vibration and Fe–O bending vibrations of the BiO_6_ and FeO_6_ octahedral groups around 532 and 420 cm^−1^, respectively, indicating the successful formation of BiFeO_3_ [48]. The BFO/PCN and U-BFO/PCN composite samples exhibit similar and a combination of spectral patterns of their constituent pristine materials.

#### 2.1.2. X-ray Diffraction (XRD)

**Figure 2:**
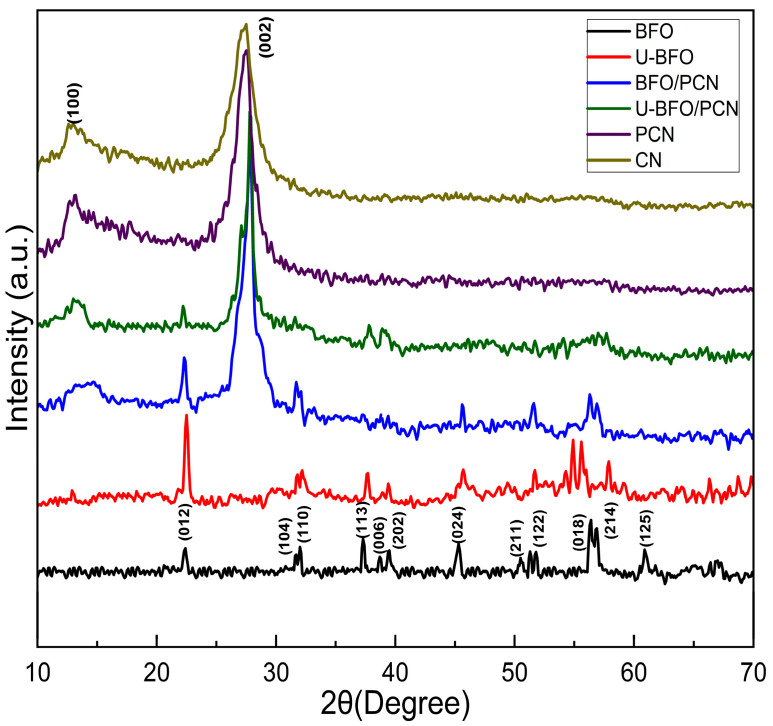
XRD patterns of pristine and composite photocatalysts.

The XRD diffraction patterns of the CN, PCN, BFO, U-BFO, BFO/PCN, and U-BFO/PCN catalyst materials are displayed in Figure 2. Two distinct characteristic diffractograms for pristine CN appeared at 27.5° and 13.1° 2θ values, which can be indexed to (0 0 2) and (1 0 0) hexagonal crystal planes (JCPDS 87-1526), respectively. The (0 0 2) diffraction peak corresponds to the interlayer spacing, while the (1 0 0) peak relates to the structural packing unit due to the in-planar repeated heptazine motifs in the CN structure [49]. The XRD patterns of PCN remain identical to those of CN, indicating that both the interlayer spacing and the basic heptazine units in CN are retained following P doping. However, an increase in the intensity of the (0 0 2) peak was observed for the PCN sample, indicating an increase in the crystallinity with P doping. In the XRD diffraction patterns of pure BFO and U-BFO catalysts, peaks corresponding to the (0 1 2), (1 0 4), (1 1 0), (1 1 3), (0 0 6), (2 0 2), (0 2 4), (2 1 1), (1 2 2), (0 1 8), (2 1 4), and (1 2 5) diffraction planes were exhibited for both materials. The lattice constants and cell volume for BFO are a = b = 5.5934 Å and c = 13.7830 Å, and a = b = 5.5867 Å and c = 13.8231 Å for U-BFO. These can be indexed as pure rhombohedral R3c BiFeO3 (JCPDS file 01-086-1518, a = b = 5.5775 Å and c = 13.8616 Å) [50]. For both the BFO/PCN and U-BFO/PCN composites, diffraction peaks of the individual catalysts are notably observed, indicating the composite formation in each case. However, there is a decrease in the intensity of the (1 0 0) diffraction peak of PCN in both composites, signaling that both BFO and U-BFO species modify the in-planar tri-s-triazine units in the CN structure. The d-spacing for CN, PCN, BFO/PCN, and U-BFO/PCN were computed using Braggs law at (002) plane of π-π stacking at 27.5°. The interlayer distance of 2.95 nm is found for the un-doped CN, while an increase in the d-spacing for P-doped CN at 3.60 nm. This is expected since phosphorous is larger than both carbon and nitrogen; thus, its incorporation could potentially expand the lattice structure of CN. The d-spacing of 5.54 and 4.35 nm were estimated for U-BFO/PN and BFO/PCN composites, indicating slight expansion from the PCN.

#### 2.1.3. N_2_ Physisorption Isotherm

**Figure 3:**
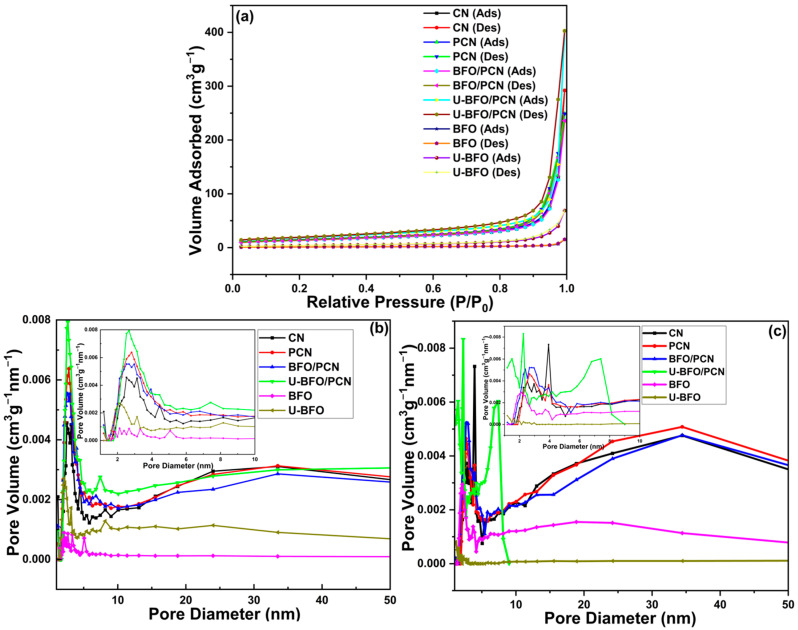
(**a**) N_2_ adsorption–desorption isotherms and BJH pore size distributions for (**b**) adsorption and (**c**) desorption curves of pristine and composite photocatalysts.

The N_2_ physisorption isotherms and pore size distribution curves of the pristine and composite materials are shown in Figure 3. As illustrated in Figure 3a, All the samples exhibited isotherms belonging to type IV isotherm with an H3 hysteresis loop as per IUPAC classification [51], indicating the development of mesoporous structure in all the materials. Generally, a larger surface area provides more accessible sites for surface reactions, which is favorable to heterogeneous catalysis [52]. The Brunauer–Emmett–Teller (BET) surface areas of CN, PCN, BFO, U-BFO, BFO/PCN, and U-BFO/PCN materials are 47.252, 51.557, 15.615, 3.893, 49.746, and 65.875 m^2^g^−1^, respectively, as shown in Table 1. The surface area of BFO is about four times larger than that of U-BFO, which could be attributed to excessive agglomeration of particles leading to morphology alteration from a flake-like shape for BFO and an elliptical-like shape for U-BFO as seen in SEM analysis. However, the synergistic surface area of U-BFO/PCN is about 1.3 times that of BFO/PCN. The Barrett–Joyner–Halenda (BJH) pore size distribution in Figure 3a (adsorption) and Figure 3b (desorption) show the samples’ combined micro/mesoporous structures, with pore widths and pore volumes displayed in Table 1. 

#### 2.1.4. Scanning Electron Microscopy (SEM)

**Figure 4:**
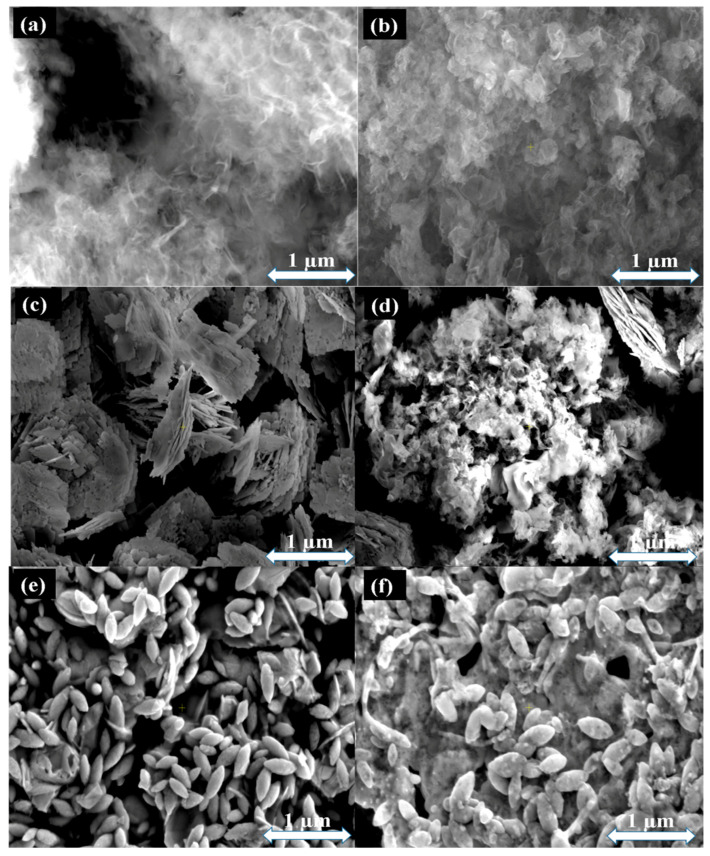

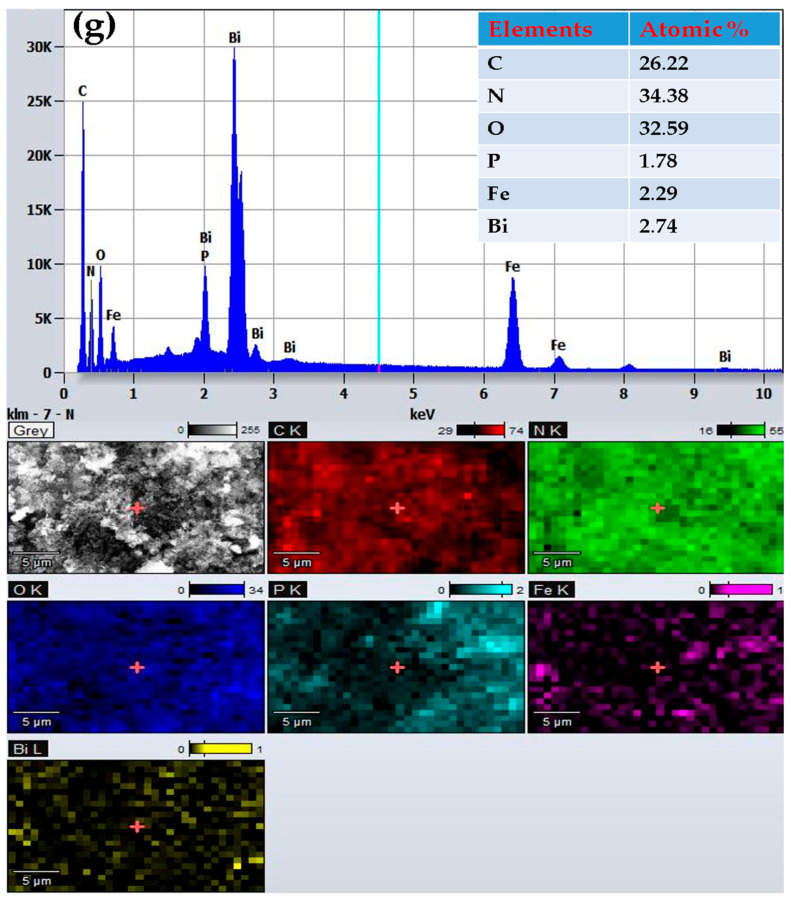
SEM images of (**a**) CN, (**b**) PCN, (**c**) BFO, (**d**) BFO/PCN, (**e**) U-BFO, (**f**) U-BFO/PCN, and (**g**) EDS spectrum and elemental mappings of U-BFO/PCN samples.

Figure 4 shows the SEM microstructural images, EDS spectra, and elemental mappings of the as-synthesized samples. The pristine and P-doped CN display a friable, non-uniform aggregated morphology, as seen in Figure 4a and Figure 4b, respectively, in consonance with Yue et al. [53]. This is a typical characteristic of a 2D porous structure owing to gas vaporization in the polymerization and carbonation processes [54]. Figure 4c exhibits an aggregated flake-like morphology for the BFO sample, which transforms into an elliptical-like shape for U-BFO, as featured in Figure 4d. For the PCN-supported composites of BFO and U-BFO, both feature morphologies similar to those of BFO and U-BFO distributed in the 2D porous network of the PCN as displayed in Figure 4e and Figure 4f, respectively, confirming the formation of BFO/PCN and U-BFO/PCN composites. Meanwhile, the presence of all the constituent elements and their composition in the composites is evidenced in the EDS spectrum for U-BFO/PCN in Figure 4g. Figure 4g displays the EDs spectrum and elemental mapping images of C, N, O, P, Fe, and Bi species for U-BFO/PCN composite heterostructure and their distributions throughout the entire selected area, which similarly confirms the successful construction of the heterostructured composites [55].

#### 2.1.5. Thermogravimetric Analysis with Derivative Thermogravimetry (TGA-dTG)

**Figure 5:**
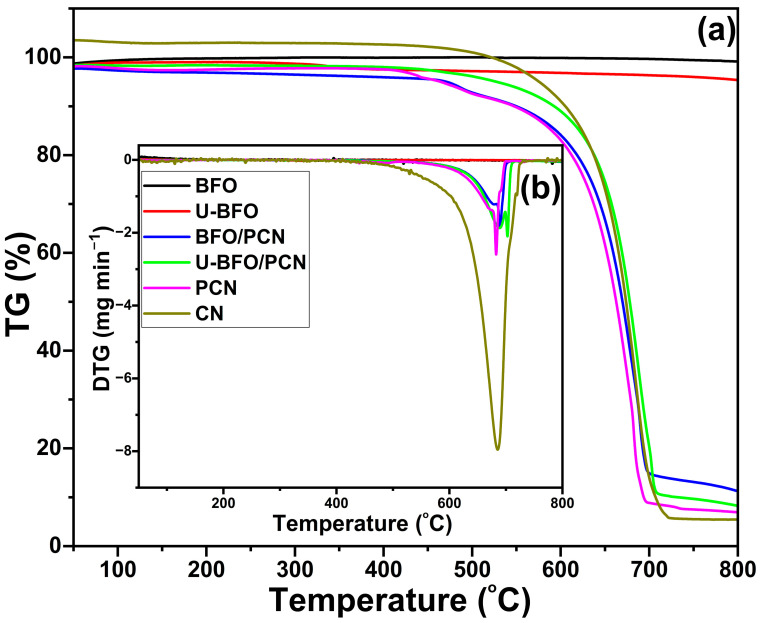
(**a**) TGA and (**b**) DTG curves of the pristine and composite samples.

Thermogravimetric (TG) (Figure 5a) and derivative thermogravimetric (DTG) (Figure 5b) curves of the as-prepared pristine and composite materials are shown in Figure 5. It can be seen that the decomposition of CN material noticeably began and completed between 550 and 722 °C, with a residual weight fraction (RWF) below 5%, indicating the stability of CN below 550 °C as previously obtained [56]. However, the decomposition of PCN started at a lower temperature but completed at the same temperature with an RWF of about 5.5%. For BFO and U-BFO samples, there is a small initial weight loss due to the release of surface adsorbed gases, with their RWF at the same temperature as high as 99.15% for BFO and 98.81% for U-BFO. A similar RWF for BFO was reported by Piña-Salazar et al. [57]. The RWF values for both composites are above 10%, signaling the integration of BFO and U-BFO in the PCN.

#### 2.1.6. Ultraviolet-Visible (UV-vis) Spectroscopy

**Figure 6:**
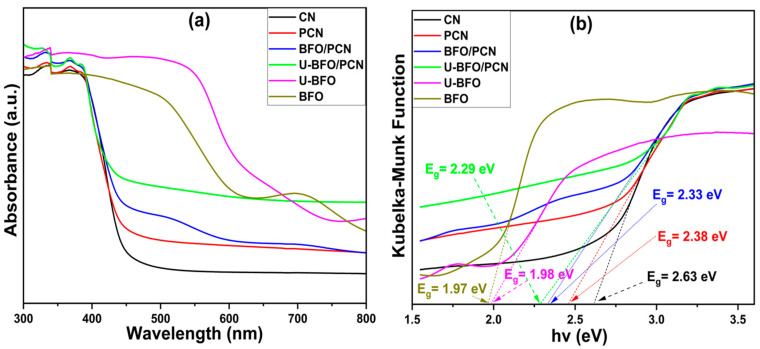
UV-vis spectra (**a**) and Tauc plot curves (**b**) of the pristine and composite samples.

Figure 6 shows the light absorption ability of all the pristine and composite samples. As can be seen in Figure 6a, all the samples exhibit apparent absorption in the visible light spectrum, and the absorption wavelengths of CN, PCN, BFO/PCN, U-BFO/PCN, BFO, and U-BFO in Figure 6a are around 471, 521, 532, 542, 629, and 627 nm, respectively. The red shift in the visible-light spectrum edges of BFO/PCN and of U-BFO/PCN composites is caused by the synergistic surface interaction between BFO, U-BFO, and PCN. From the Tauc plot in Figure 6b, the band gap energies of CN, PCN, BFO, U-BFO, BFO/PCN, and U-BFO/PCN are approximately computed as 2.63, 2.38, 2.33, 2.29, 1.98, and 1.97 eV, respectively. The light absorption properties of both composites reveal improved visible light absorption ability, suggesting they can show an enhanced photocatalytic performance.

#### 2.1.7. Photoluminescence Study

**Figure 7:**
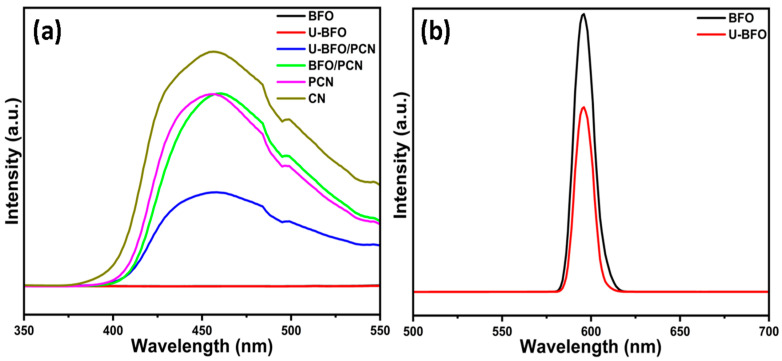
PL spectra (**a**) showing the emission of all the catalysts and (**b**) showing the emissions of BFO and U-BFO samples.

Figure 7 displays the photoluminescence (PL) spectra of all the pristine and composite materials to further elucidate their electronic structure and evaluate the interfacial separation and transfer of photo-generated charges. From Figure 7a, a strong emission peak at 456 nm is observed for CN, which slightly decreased for P-doped CN. The U-BFO/PCN composites show a weaker emission intensity than both CN and PCN, indicating a significant deceleration of charge carrier recombination compared to the CN and PCN systems [58]. However, the spectra of BFO/PCN composites did not show any noticeable emission reduction compared to that of PCN, which further could explain why it was outperformed by the heterojunction effect of the U-BFO/PCN composites in the RhB degradation. No emission peak was recorded for BFO and U-BFO until 591 nm, and their spectra in Figure 7b show that the photogenerated charge carriers in BFO have more chance of recombining than those of the U-BFO sample [59]. The PL results prove that an efficient formation of U-BFO/PCN heterojunction could stimulate the separation and transfer of charge carriers by slowing their recombination, thus improving photocatalytic RhB degradation.

### 2.2. Photodegradation of RhB Evaluation

**Figure 8:**
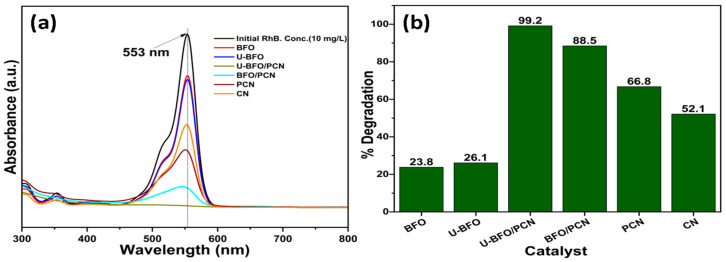
Absorbance spectra (**a**) and degradation efficiencies (**b**) of the pure and composite catalysts after 20 min of photodegradation.

The photocatalytic activity of all the pristine and binary composite heterostructures was analyzed via visible light degradation of aqueous RhB. There is a marginal reduction in the absorption peaks of the RhB dye solution, demonstrating that the equilibrium between adsorption and desorption has been reached within 30 min of the dark reaction. However, the light-induced reactions exhibit a substantial decline in the peak absorbance of RhB, indicating the successful photodegradation of RhB. Figure 8 represents the spectral absorption profiles and degradation efficiencies of the pure and binary composite catalysts for comparison in their photocatalytic efficacy. Figure 8a vividly illustrates that the UV-visible absorption spectrum prominently features a peak absorption at approximately 553 nm. Consequently, we have selected the absorbance at this specific wavelength to calculate both the percentage degradation and degradation rate of RhB, taking into account the decline in intensity. Figure 8b shows that both composites demonstrate excellent photocatalytic activity against RhB, displaying photodegradation efficiencies of 99.2% for U-BFO/PCN and 88.5% for BFO/PCN following just 20 min of degradation. However, for the bare BFO, U-BFO, and PCN catalysts, the recorded efficiencies only reach 25.5, 29.3, and 73.9%, respectively, after the same irradiation time. Furthermore, RhB photodegradation rate constants are calculated via a linearized pseudo-first-order reaction kinetics plot obtained from the quantitative time-dependent data. As revealed in Table 2, the U-BFO/PCN composite demonstrates a pseudo-first-order rate constant of 2.41 × 10^−1^ min^−1^, 3.9 times, 54 times, and 6.3 times more than BFO/PCN (1.08 × 10^−1^ min^−1^), U-BFO, (4.50 × 10^−3^ min^−1^), and PCN, (6.72 × 10^−2^ min^−1^), respectively. 

**Figure 9:**
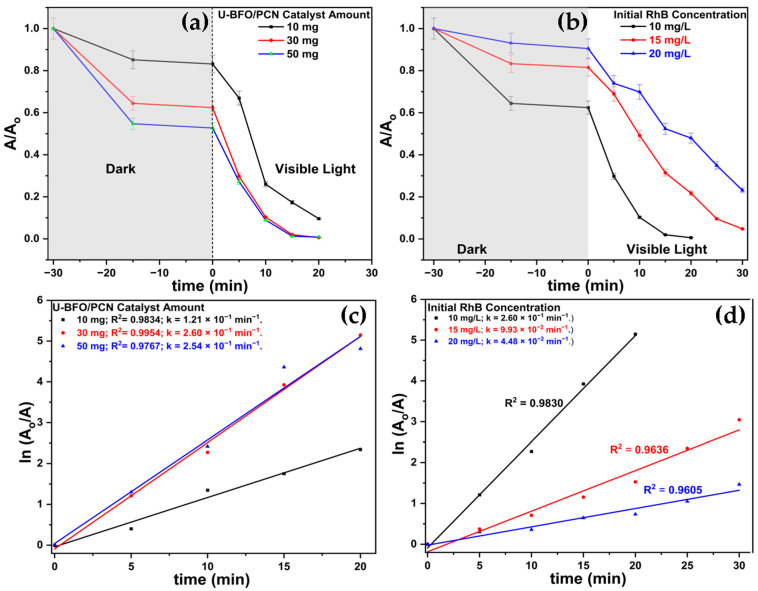
Photodegradation of varied amounts of U-BFO/PCN composites (**a**), varied initial RhB concentration (**b**), and their corresponding linearized pseudo-first-order plots (**c**,**d**).

The efficacy of the U-BFO/PCN composite was further investigated to optimize the initial dye concentration and catalyst quantity for RhB degradation. This involved varying the catalyst mass and the concentration of the dye pollutant while maintaining the solution’s pH at 6. Figure 9 illustrates the absorbance spectra, showcasing the impact of initial RhB concentration and catalyst quantity using the U-BFO/PCN composite. Figure 9a depict variations in catalyst quantity, while Figure 9b demonstrate changes in the initial concentration of RhB, with their corresponding time-dependent and pseudo-first-order plots in Figure 9c,d. The results illustrate that the degradation rate of RhB (10 mgL^−1^) rises as the photocatalyst amount increases from 10 mg to 30 mg, but it slightly decreases for 50 mg. The escalation in degradation rate, from 0.121 min^−1^ for 10 mg catalyst dosage to 0.260 min^−1^ for 30 mg, can be attributed to the enhanced reaction kinetics facilitated by additional active sites, allowing more RhB molecules to interact with the catalyst [60]. Conversely, employing 50 mg of the catalyst resulted in a reduced degradation rate of 0.228 min^−1^, indicating catalyst aggregation leading to diffusion limitation and reduced effective surface area [61]. Therefore, 30 mg of U-BFO photocatalyst was identified as the optimal quantity for the photocatalytic reaction. In examining the impact of initial concentration, three concentrations of RhB (10, 15, and 20 mgL^−1^) were utilized, with the catalyst amount and pH held constant at 30 mg and 6, respectively. Figure 9d reveals an inverse relationship between the initial RhB concentration and the pseudo-first-order rate constant, evidenced by the decrease in degradation rates from 0.260 min^−1^ to 0.0448 min^−1^ as the initial RhB concentration rises from 10 to 20 mgL^−1^. This suggests a potential constraint in light penetration and challenges in RhB molecules accessing the active sites of the U-BFO/PCN catalyst [62].

The results of the photocatalytic degradation of dyes for some photocatalysts are shown in Table 3. It can be seen that the combination of U-BFO and PCN and that of BFO and PCN demonstrated better photocatalytic efficacy than previously published works. 

#### 2.2.1. Free-Radical Scavenging

**Figure 10:**
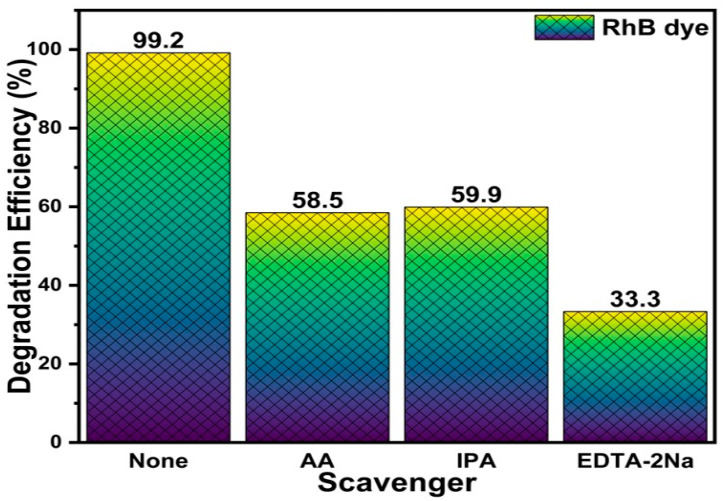
Scavenger tests for RhB degradation using U-BFO/PCN composites.

To study the degradation mechanism of U-BFO/PCN, several radical scavengers, namely ascorbic acid (AA), isopropyl alcohol (IPA), and ethylenediaminetetraacetic acid (EDTA) were used in degradation experiments to capture the holes (^•^O_2_^−^), hydroxyl (^•^OH), and superoxide (h^+^) radicals [72], thus their reaction performance was investigated and illustrated in Figure 10. It can be observed that from the addition of the three mentioned scavengers, EDTA has the most effect on RhB degradation under visible light irradiation because it shows a drastic decrease in the degradation efficiency, with IPA and EDTA following. This signifies that photogenerated holes have a crucial role in the degradation of RhB over U-BFO/PCN composites. However, the superoxide and hydroxyl radicals also significantly affect the degradation reaction.

#### 2.2.2. Proposed RhB Degradation Mechanism

**Figure 11:**
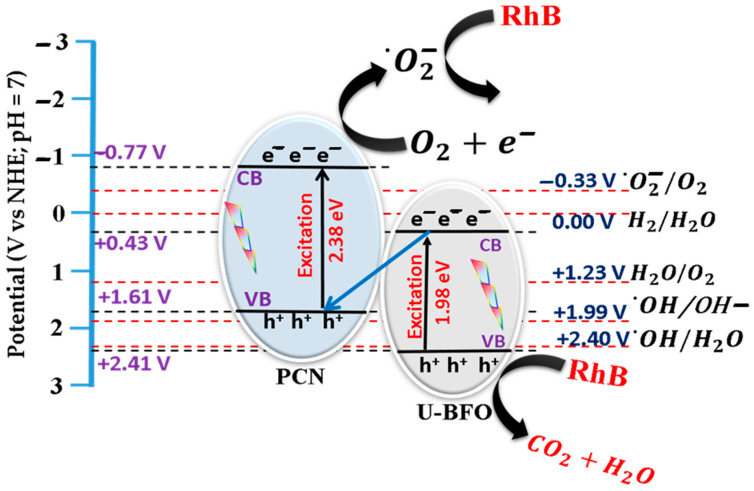
Proposed mechanism for RhB degradation over U-BFO/PCN heterojunction.

In the scavenger test, in combination with the band structure analysis results of U-BFO and PCN, the improved mechanism in photocatalytic RhB degradation behavior of U-BFO/PCN heterojunctions was proposed in Figure 11. Equations (1) and (2) below were used to compute the VB edge potential (EVB) and CB edge potential for both U-BFO and PCN [73], which, together with the scavenger test for ion radicals, the mechanism in photocatalytic RhB degradation behavior of U-BFO/PCN heterojunction was proposed in Figure 10.

(1) $$E_{VB}=X-E^{e}+0.5E_{g}$$

(2) $$E_{CB}=E_{VB}-E_{g}$$

where *E_VB_* and *E_CB_* represent the energies of the valence band and conduction band edges, *X* denotes the absolute electronegativity of materials (X_U-BFO_ = 4.92 eV and X_PCN_ = 4.92 eV), while *E^e^* stands for the energy of free electrons vs. hydrogen (4.5 eV), and *E_g_* is the band gap energy of the catalysts. The computed CB and VB edges of PCN and U-BFO are −0.77 eV and +1.61 eV and +0.43 eV and +2.41 eV, respectively. 

The *E_VB_* of PCN (+1.66 eV) is less than the potentials of ^•^OH/H_2_O (+2.40 V vs. NHE) and ^•^OH/OH- (+1.99 V vs. NHE), and the reduction potential of the photogenerated electrons from the CB of U-BFO (+0.43 V vs. NHE) cannot meet the thermodynamic requirements for reducing O_2_ (−0.33 V vs. NHE) to ^•^O_2_^−^ [74,75]. This ruled out the conventional type-II mechanism since it would require the holes in the CB of PCN and the ^•^O_2_^−^ produced by the photogenerated electrons in the CB of U-BFO to perform the photodegradation reaction. As PCN possesses higher CB and VB positions than U-BFO, we proposed a direct Z-scheme heterojunction, in which the photogenerated electrons from the CB of U-BFO recombine with the holes in the VB of PCN due to the in-built electric field. Consequently, the photo-induced electrons from the CB of PCN reduced O_2_ to ^•^O_2_^−^ radicals, which, together with the holes in the VB of U-BFO, proceeded with the RhB photodegradation maintaining a strong redox and effective e-/h+ pairs separation [76].

#### 2.2.3. Recyclability of the Binary Catalysts

**Figure 12:**
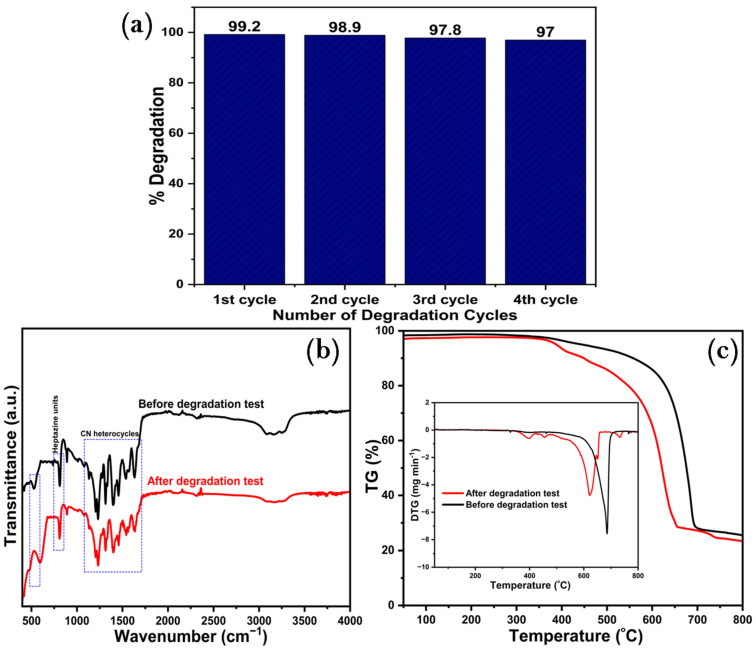
(**a**) Degradation runs (**b**) FT-IR spectra of U-BFO/PCN before and after the degradation experiment and (**c**) TG/DTG profiles of U-BFO/PCN before and after the degradation experiment.

Figure 12 displays the reusability results of the two binary composite photocatalysts obtained, with photooxidation efficiency varied across 97% and 99.2% in four cycles of photodegradation experiments to ascertain their photocatalytic stability. Figure 12a demonstrates that U-BFO/PCN maintained outstanding photocatalytic efficiency (97%) toward RhB degradation after the fourth cycle, indicating its excellent photostability. Figure 12b displays the FT-IR spectra of the binary composite before the degradation and after the fourth cycle of the experiment. The results ascertain that the composite maintained its spectral peaks relative to the as-synthesized ones, corroborating their exceptional recyclability test results. The TG/DTG profiles of the heterostructured composites in Figure 12c also did not change significantly, thus further affirming that U-BFO/PCN exhibited excellent photostability and reusability for practical applications.

## 3. Materials and Methods

### 3.1. Materials and Reagents

Urea powder (CH_4_N_2_O; 60.06 g/mol, ≥99.5%) was procured from Carl Roth GmbH (Karlsruhe, Germany) as the precursor for the synthesis of g-C_3_N_4_ and urea-assisted synthesis of BiFeO_3_, while diammonium hydrogen phosphate [(NH_4_)_2_HPO_4_; 132.07 g/mol, 98%] purchased from Reactivul Plus S.R.L (Bucharest, Romania) was used as a phosphorous source in the preparation of P-doped g-C_3_N_4_. Bismuth (III) nitrate pentahydrate (Bi (NO_3_)_3_·5H_2_O; 485.07 g/mol, ≥98%), and iron(III) nitrate nonahydrate (Fe (NO_3_)_3_·9H_2_O; 404 g/mol, ≥98%) procured from Sigma Aldrich (Darmstadt, Germany) were employed as the precursor salts for the fabrication of perovskite BiFeO_3_. Methyl alcohol (CH_3_OH; 32.04 g/mol, 99.85%) and ethyl alcohol (C_3_H_2_OH; 46.07 g/mol, 99.5%) were acquired from Chemical Ch-C (Iasi, Romania). All chemical reagents were used as acquired without additional treatment.

### 3.2. Synthesis of P-Infused g-C_3_N_4_

A modified polycondensation synthesis route reported by Lin et al. [77] was adopted for the infusion of phosphorous in the bulk structure of as-obtained g-C_3_N_4_ prepared via thermal polymerization of urea powder [78]. In a typical experiment, 20 g of urea powder covered in a porcelain crucible was placed in a muffle furnace and calcined first at 550 °C for 1 h with a ramp rate of 10°/min from room temperature, followed by sustained heating at the same temperature for 3 h. A fine, yellow-colored g-C_3_N_4_ powder was obtained by pulverizing the resultant friable agglomerated. Thereafter, NaPO_2_H_2_.H_2_O and as-pulverized g-C_3_N_4_ in 1:5 ratios were dispersed in 40 mL methyl alcohol and ultrasonicated at 30 °C for 30 min to obtain a PCN catalyst. The pre-sonicated blend was then magnetically stirred at 80 °C until complete volatilization of the solvent was attained. Subsequently, the sample was dried at 80 °C for 12 h and calcined at 550 °C for 2 h. The as-obtained PCN sample was then pulverized and stored for analytical characterizations and experimentation.

### 3.3. Urea-Aided Synthesis of BiFeO_3_

A urea-aided BiFeO_3_ (U-BFO) ellipsoids were successfully achieved through a subtly adjusted hydrothermal technique reported by Wei et al. [79]. Typically, 0.008 mol Bi (NO_3_)_3_·5H_2_O, 0.008 mol Fe (NO_3_)_3_·9H_2_O, and 0.2 mol CO(NH_2_)_2_ were dissolved in double distilled water to prepare a 50-mL homogeneous solution. The mixture was magnetically stirred continuously with the addition of 4 mL HNO_3_ until a homogeneous suspension was achieved. To the ensuing dispersion, a 10 M KOH solution was then added dropwise under robust magnetic agitation until it changed into a clear and brownish cocoa-infused blend. The mixture was subsequently transferred into a 100-mL Teflon-lined autoclave reactor and hydrothermally treated at 120 °C for 16 h. The reactor was allowed to cool naturally before a solid, brown-powdered powder was collected after severally washing the as-obtained sample with double distilled water and ethyl alcohol via centrifugation at 2000 rpm. The as-collected brown powder was then dried at 80 °C for 12 h, calcined at 550 °C for 2 h, and labeled as U-BFO before storing for analytical characterizations and further experimentation. Urea-free BiFeO_3_ (BFO) flakes were synthesized using the same methodology without urea addition in the blend.

### 3.4. Construction of P-g-C_3_N_4_/BiFeO_3_ Heterojunction

In the construction of PCN/BFO or PCN/U-BFO heterojunctions, different weight percentages (5, 10, and 15 wt. %) of BFO or U-BFO relative to PCN were prepared. Typically, a 10 wt. % BFO/PCN or U-BFO/PCN composite was fabricated by dispersing 0.10 g BFO or U-BFO and 0.90 g PCN in 45 mL CH_3_OH and ultrasonicated for 30 min at 30 °C to obtain a homogeneous blend, followed by uninterrupted agitation of the blend at 80 °C until complete evaporation of the solvent was achieved. Other weight percentages (5 and 15 wt. %) were fabricated using the same procedure by weighing the appropriate masses of BFO/U-BFO and PCN materials. The as-fabricated heterostructured composites were calcined at 550 °C for 2 h before storing for analytical investigations and photocatalytic degradation studies.

### 3.5. Materials Characterizations

The crystalline structures of the bare BFO, U-BFO, and PCN, as well as PCN/BFO and PCN/U-BFO composites, were analyzed by powder X-ray diffraction (XRD) and Fourier-transform infrared (FT-IR) spectroscopy to acquire insights on their phase, crystallographic structures, and chemical composition. XRD diffractograms were scanned in 2θ analysis range of 10 to 80° at a 5°/min scan rate employing a Bruker D8 Advance diffractometer (Karlsruhe, Germany; θ-θ type, Cu-Kα radiation (λ = 1.5418 Å), 40 kV, and 40 mA). The identification of FT-IR spectra of all the samples was discerned in the spectrum scanning range of 400–4000 cm^−1^ with a Nicolet Shimadzu IRTracer-100 FT-IR spectrophotometer (Kyoto, Japan). The textural properties were analyzed by N_2_ physisorption isotherm under 77 K using a Quantachrome Nova 2200e (Boynton Beach, FL, USA) instrument, with all the samples outgassed at 200 °C for 4 h before the physisorption analysis. A ThermoFisher Scios 2 HIVAC Dual-Beam FIB-SEM (Brno, Czech Republic) instrument was applied to monitor the morphologies. A Setaram Labsys Evo S60/58986 TG analyzer (Burladingen, Germany) was employed for the TGA-DTA investigation. The measurements were made in a sustained argon gas flow from 30 to 800 °C with a 10 °C min^–1^ temperature gradient. The optical spectra of the samples were investigated by a UV–vis spectrophotometer (Jasco UV-Vis V-550, Tokyo, Japan) in a 200 to 800 nm wavelength range. The photoluminescence (PL) spectra of the samples are studied on a Shimadzu RF 6000 spectrofluorophotometer (Kyoto, Japan).

### 3.6. Photocatalytic Degradation of RhB

The photodegradation performance study of the pure and composite samples was carried out towards degradation of aqueous rhodamine B (RhB) under visible light illumination in a Toption photochemical reactor (Xi’an, China) equipped with a long arc Xe lamp with an emission wavelength of λ > 400 nm. Typically, 30 mg of photocatalyst is dispersed in a 50 mL aqueous solution of 10 mg/L RhB dye in a glass tube. The tube was then put in the reactor, and the solution was continuously agitated in the dark for 30 min to achieve adsorption–desorption balance. Subsequently, the degradation proceeded with visible light illumination, and a few mL of the suspension was collected at different intervals to measure the maximum absorbance at 553 nm for RhB using a Shimadzu 3600iPlus UV–vis spectrophotometer (Columbia, MD, USA). At the end of each degradation cycle, the photocatalyst is centrifuged at 2000 rpm for 5 min to recover the sample for recycling. The efficiency of RhB degradation (E,%) was calculated using Equation (3) [80]. At the same time, the rate of RhB photodegradation was assumed to obey the popular pseudo-first-order kinetics, and the rate constant, k, for photodegradation, was computed from the first-order relation (Equation (4)).

(3) $$\mathrm{Efficiency}\;\left(E,\%\right)=\frac{A_{0}-A}{A_{0}}\times 100$$

(4) lnA0A=kt

where k, A_0_, and A are the pseudo-first-order rate constant, the absorbance at the initial concentration of aqueous RhB solution, and the absorbance at the concentration of RhB at any specific time of visible-light irradiation, respectively.

## 4. Conclusions

In conclusion, phosphorous-doped graphitic carbon nitride (PCN) supported BFO composites with distinctive morphological texture and electronic configuration have been proficiently constructed for the degradation of RhB. The mono-doping of phosphorous into the bulk structure of CN improved visible-light absorption by diminishing the optical bandgap and enriching the surface density of active binding sites. The excellent photodegradation activity of U-BFO/PCN composites was brought by the reduced diffusion distance of charge carriers to the surface. This promotes the separation and redox capacity of the catalyst due to synergistic impacts of both U-BFO and PCN catalysts, as well as the morphologically modified BFO configuration with the introduction of urea in its synthesis. Thus, this approach is facile for fabricating nonmetal-atoms-infused CN composites incorporating oxide photocatalysts with modified electronic and morphological structures. Moreover, owing to its improved structure, the PCN-supported U-BFO composite holds considerable potential for application in diverse environmental remediation, including photocatalytic degradation of industrial dye effluents.

## Figures and Tables

**Table 1:** BET-specific surface area, pore volume, and pore diameter of CN, PCN, BFO, U-BFO, BFO/PCN, and U-BFO/PCN samples.

Catalyst	BET SSA (m^2^/g)	Pore Size Distribution (Adsorption)					
		Pore Diameter (nm)			Pore Volume (m^3^/g)		
		Ads.	Des.	Avr.	Ads.	Des.	Avr.
BFO	15.615	2.130	2.127	2.129	0.024	0.109	0.133
U-BFO	3.893	2.124	1.188	1.656	0.108	0.022	0.065
BFO/PCN	49.746	2.525	2.661	2.593	0.563	0.365	0.464
U-BFO/PCN	65.875	2.664	3.943	3.304	0.619	0.621	0.620
PCN	51.557	2.814	4.497	3.341	0.582	0.383	0.483
CN	47.252	2.523	3.933	3.228	0.448	0.450	0.449

**Table 2:** Efficiency, pseudo-first-order rate constant, and linear regression coefficient of RhB degradation.

Catalyst	Efficiency (%)	Rate Constant, k (min^−1^)	R^2^
BFO	23.8	0.0031	0.9621
U-BFO	26.1	0.0042	0.9733
BFO/PCN	88.5	0.1080	0.9785
U-BFO/PCN	99.2	0.2410	0.9870
PCN	67.4	0.0560	0.9784
CN	52.1	0.0368	0.9755

**Table 3:** Comparison of some g-C_3_N_4_-based heterostructure for photodegradation of dyes aqueous media.

Catalyst (Amount, mg)	Conditions	Efficiency (%)-Rate Const. (min^−1^)	Ref.
gC_3_N_4_/TiO_2_/kaolinite (200 mg)	100 mL (CIP 10 mg/L)—visible light 300 W	92%—0.008	[63]
SiO_2_/gC_3_N_4_ (10 mg)	100 mL (XO 10 ppm; AO, 10 ppm)—visible light	XO; 84%—0.014 AO; 70%—0.009	[64]
WO_3_/g-C_3_N_4_ (100 mg)	100 mL (RhB 100 mg/L)—visible light 500 W	96%—0.063	[65]
BiFeO_3_/g-C_3_N_4_ (50 mg)	25 mL (RhB 40 mg/L)—natural sunlight	96%—0.039	[66]
CuWO_4_/g-C_3_N_4_ (50 mg)	50 mL (RhB 50 mg/L)—visible light 300 W	93%—0.015	[67]
MgO@g-C_3_N_4_ (50 mg)	50 mL (IC 25 ppm)—visible light	99%—0.084	[68]
Cu-ZnO/gC_3_N_4_ (50 mg)	100 mL (IC 10 mg/L)—visible light	98%—0.088	[69]
CdMoO_4_/g-C_3_N_4_ (50 mg)	50 mL (MB 10 ppm)—visible light	98%—0.020	[70]
V_2_O_5_/protonated g-C_3_N_4_ (25 mg)	100 mL (MB 10 mg/L)—sunlight	94%—0.024	[71]
U-BiFeO_3_/P-g-C_3_N_4_ (30 mg)	50 mL, (RhB 10 mg/L)—visible light 500 W	99%—0.260	This work

## Data Availability

Data will be made available upon request.
